# Effects of Plant Crown Shape on Microwave Backscattering Coefficients of Vegetation Canopy

**DOI:** 10.3390/s21227748

**Published:** 2021-11-21

**Authors:** Xiangchen Liu, Yun Shao, Long Liu, Kun Li, Jingyuan Wang, Shuo Li, Jinning Wang, Xuexiao Wu

**Affiliations:** 1 Aerospace Information Research Institute, Chinese Academy of Sciences, Beijing 100101, China; liuxc@radi.ac.cn (X.L.); shaoyun@aircas.ac.cn (Y.S.); liulong@aircas.ac.cn (L.L.); wangjingyuan19@mails.ucas.ac.cn (J.W.); lishuo191@mails.ucas.ac.cn (S.L.); 2 College of Resources and Environment, University of Chinese Academy of Sciences, Beijing 100049, China; 3 Laboratory of Target Microwave Properties (LAMP), Deqing Academy of Satellite Applications, Huzhou 313200, China; wxx@dasa.net.cn; 4 Beijing Mcfly Technology Co., Ltd., Beijing 100101, China; 5 College of Geodesy and Geomatics, Shandong University of Science and Technology, Qingdao 266590, China; wjn2018sk@gmail.com

**Keywords:** crown shape, microwave backscattering coefficients, Tor Vergata Model, computational electromagnetics

## Abstract

A microwave scattering model is a powerful tool for determining relationships between vegetation parameters and backscattering characteristics. The crown shape of the vegetation canopy is an important parameter in forestry and affects the microwave scattering modeling results. However, there are few numerical models or methods to describe the relationships between crown shapes and backscattering features. Using the Modified Tor Vergata Model (MTVM), a microwave scattering model based on the Matrix Doubling Algorithm (MDA), we quantitatively characterized the effects of crown shape on the microwave backscattering coefficients of the vegetation canopy. FEKO was also used as a computational electromagnetic method to make a complement and comparison with MTVM. In a preliminary experiment, the backscattering coefficients of two ideal vegetation canopies with four representative crown shapes (cylinder, cone, inverted cone and ellipsoid) were simulated: MTVM simulations were performed for the L (1.2 GHz), C (5.3 GHz) and X (9.6 GHz) bands in fully polarimetric mode, and FEKO simulations were carried out for the C (5.3 GHz) band at VV and VH polarization. The simulation results show that, for specific input parameters, the mean relative differences in backscattering coefficients due to variations in crown shape are as high as 127%, which demonstrates that the crown shape has a non-negligible influence on microwave backscattering coefficients of the vegetation canopy. In turn, this also suggests that investigation on effects of plant crown shape on microwave backscattering coefficients may have the potential to improve the accuracy of vegetation microwave scattering models, especially in canopies where volume scattering is the predominant mechanism.

## 1. Introduction

The backscattering behavior of vegetation targets is important to consider when conducting parameter inversion in remote sensing applications. A microwave scattering model is a powerful tool that can be used to analyze the backscattering characteristics of vegetation in a Synthetic Aperture Radar (SAR) image. Moreover, the accuracy of a microwave remote sensing inversion model is inferior to that of a microwave forward scattering model. Based on microwave scattering theory, the microwave scattering model of vegetation is used to establish a functional relationship between vegetation parameters and radar backscattering coefficients. In general, the vegetation microwave scattering model can be grouped into two parts depending on whether it is coherent or not [1]. The incoherent model is based on the radiation transfer theory, and the coherent model relies on the analytical wave theory. In the past 40 years since the Water Cloud Model (WCM) was proposed in 1978 [2], many scattering models of vegetation have been developed, such as the Michigan Microwave Canopy Scattering Model (MIMICS) [3], the Branching Model (BM) [4], the Fully Phase-Coherent Model (FPCM) [5], the Tor Vergata Model (TVM) [6], etc.

To develop a high-fidelity scattering model, it is critical to use a mathematical equation that describes a realistic vegetation scene, which requires the consideration of a large number of morphological parameters. Crown shape is one of the basic parameters used in forestry, and the extraction of the crown shape is important in forest resource management [7,8,9]. Crown shape is an important factor affecting the spatial heterogeneity of the vegetation canopy, which in turn affects the backscattering characteristics of the vegetation. In remote sensing applications, Nelson observed that different crown shapes used in a parameter estimation model could result in variations of nearly 25% in the estimates of canopy height derived from airborne laser data [10]. When inverting tree structural parameters, the wrong hypothesis regarding the crown shape has caused deviations as high as 250% [11]. For optical data, the choice of crown shape affected the estimation of the emissivity of grass [12]. Therefore, the relationship between crown shape and radar backscattering coefficients is an important consideration in the development of microwave scattering models and related applications.

The crown shape is a surface that envelops individual plants and confines the location of scattering elements within the vegetation canopy. It also indirectly affects simulated backscattering coefficients, especially for coherent scattering models. However, only a few models include defined crown shapes in their simulations. In general, an implied or hidden crown shape is used. Depending on the scattering medium, microwave scattering models can be classified into continuous and discrete models. The Water Cloud Model is a classical continuous model. The assumption that the dielectric water cloud comprises identical water particles that are uniformly distributed throughout the medium implies that the crown shape is based on the Radiation Transfer Equation (RTE), which generally treats the canopy as a stack of horizontal layers [13,14]. The WCM allows interpretation of some experimental results obtained by scatterometers, but its simplicity limits the application to get an insight into vegetation scattering [1,6]. The crown shape and plant spacing, as well as other morphological parameters, are hidden behind the statistical distribution of the scatterers. A few studies have included defined crown shapes; for instance, ellipsoidal and cylindrical crown shapes were used in a coherent scattering model of Sahelian grassland [15], and two truncated cones were used to simulate the crown shape of a mangrove forest [16]. To our knowledge, there are few numerical models or methods to describe the relationships between crown shapes and backscattering features of vegetation canopies.

This paper is devoted to developing a method to analyze the effects of crown shape based on one of the microwave scattering models. The Tor Vergata Model, which is based on the Matrix Doubling Algorithm, is extensively used in vegetation modeling and has been validated using radiometer data for forests [17], and a satisfactory agreement using SAR data was observed between simulated and experimental results over sunflower fields [6]. In a 3D forest backscattering simulation, the TVM was introduced to improve the estimate of the canopy volume scattering [18], and the results showed that the TVM could promote the multiple scattering estimate of vegetation canopy. Therefore, the TVM was selected and modified for our research, and Liu simulated the difference between the TVM and MTVM results for the inverted cone canopy of VV polarization at 5.3 GHz, which was −3.4 dB and indicated that the crown shape may have an influence on vegetation backscattering behavior [19].

However, ideal vegetation canopies that have different crown shapes but identical canopy parameters are rare in nature, making it difficult to carry out experimental measurements for vegetation canopies. Hence, an electromagnetic numerical method was also implemented in an attempt to model and simulate vegetation canopies with different crown shapes. For this purpose, we used FEKO, which is a comprehensive computational electromagnetic tool that is used to calculate the 3D electromagnetic field with numerical methods. The calculation results obtained with FEKO have high reliability and have been validated in many applications [20], but few researchers have conducted FEKO simulations for the backscattering characteristics of natural objects such as vegetation [21]. In this study, FEKO simulation was used for 3D canopy modeling and backscattering simulation of vegetation canopies with different crown shapes. The results establish a good foundation for exploring computational electromagnetic methods in the microwave scattering domain of vegetation.

In Section 2, four representative different vegetation crown shapes are modeled, and the conventional Tor Vergata Model is modified to include the crown shape effect. After that, the modeling and FEKO simulations of 3D vegetation canopies with the same crown shapes are described. In Section 3, we present the simulation results and summarize some basic rules concerning the relationship between crown shapes and backscattering coefficients. Conclusions are provided in Section 4.

## 2. Models and Methods

### 2.1. The Modeling of Crown Shape

**Figure 1:**
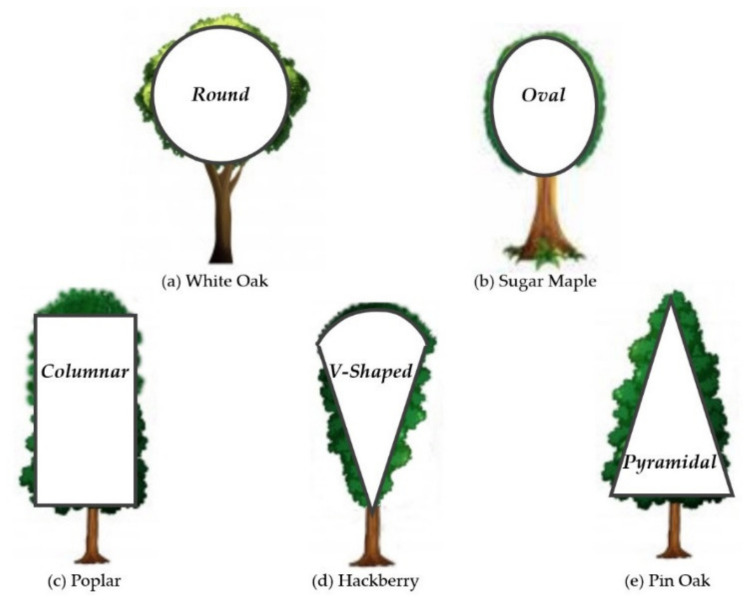
Crown shapes in nature [22]. Vegetation crown shapes are written in bold; the corresponding typical vegetation types are written in normal font and are: (**a**–**e**).

Various kinds of vegetation and crop crown shapes exist in nature, such as round, oval, columnar, V-shaped and pyramidal shapes, among others. Figure 1 shows the five mentioned different crown shapes and the corresponding typical vegetation.

As a rule, vegetation is divided into several independent layers in microwave scattering modeling. For example, a forest is usually divided into three regions: (a) the crown region, (b) the trunk region and (c) the underlying ground region. A crop is composed of the crown region and the underlying ground region, and it also includes the grain region at harvest time. The canopy region, which is a mixed layer of various vegetation components (leaf, branch, stalk, etc.), is the most complex sub-layer in a vegetation scene. Many parameters are required to describe a vegetation canopy in detail.

**Figure 2:**
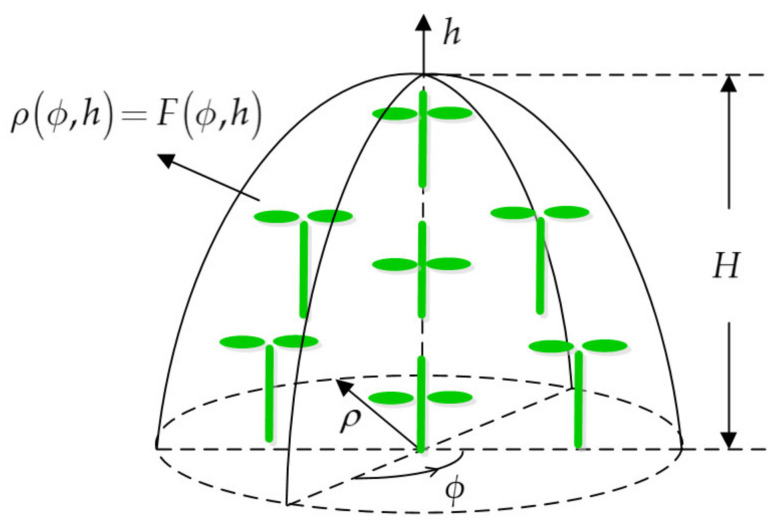
Tridimensional geometry representation of the ideal crown shape in the cylindrical coordinate system $\left(\rho ,\;\phi ,\;h\right)$, where an ellipse represents a leaf, and a cylinder represents a branch; H is crown height, and ρ(ϕ, h)=F(ϕ, h) is the crown envelope equation.

The crown shape is a surface that surrounds individual plants and envelops all of the vegetation components within the canopy. As shown in Figure 2, the envelope surface in a cylindrical system can be expressed as

(1) ρ(ϕ, h)=F(ϕ, h), ϕ ∈ [0, 2π), h ∈ [0, H],

where ϕ is the azimuthal angle, h is the vertical height, H is the crown height, and ρ(ϕ, h) is the radius at a specified position; the radius is a function of ϕ and h. In the crown envelope surface, an ellipse represents a leaf, and a cylinder represents a branch.

In particular, the crown shape can be treated as an envelope surface that is derived from rotation about the *h*-axis by the curvilinear function F(h). The rotating surface can be expressed as

(2) ρ(h)=F(h), h ∈ [0, H],

in the cylindrical coordinate system.

To ensure that the crown shape was the only variable in modeling the vegetation canopy, an ideal vegetation canopy model was built based on the following assumptions.

- The vegetation canopy comprises a mixture of leaves and branches (or stalks), and there is no obvious stratification inside the vegetation canopy.
- Without loss of generality, we assumed that the volume density of all vegetation components in the canopy is the same. The volume density is denoted as $\rho_{v}$, and its unit is cm^−3^.
- For a given vegetation canopy, all vegetation components are uniformly distributed.
- Among the same kind of vegetation component in the canopy, the geometrical features and the physicochemical properties, such as the geometrical shape, the size and the moisture of the component, remain the same.
- In order to simplify the representation of the vegetation canopy, all leaves are horizontally arranged, and all branches or stalks are vertically aligned inside the crown envelope surface.

**Figure 3:**
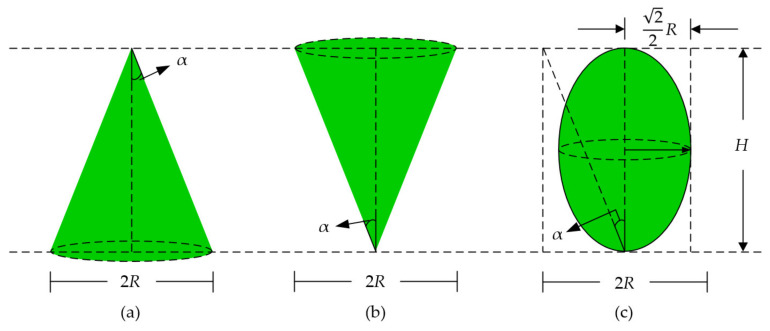
Geometric representations of the three specific crown shapes. The crown shapes, from left to right, are (**a**) cone, (**b**) inverted cone and (**c**) ellipsoid.

To investigate the effect of the crown shape on backscattering coefficients, the three ideal envelope surfaces shown in Figure 3 were considered. The origins of coordinates lie at the central point of the bottom surface or the bottom vertex. The following envelope equations correspond to the cone, the inverted cone and the ellipsoid, respectively.

(3) ρ(h)=(H−h) tanα,

(4) ρ(h)=h tanα,

(5) $$\rho (h)=\sqrt{2h(H-h)}\;\tan\alpha ,$$

where α is the cone angle, which is assumed to be α=45° in this paper. The volumes of the three ideal vegetation canopies were set to be equal. Then, the semi-axis length of the ellipsoid can easily be obtained as

(6) $$R^{\prime }=\frac{\sqrt{2}}{2}.$$

### 2.2. The Modified Tor Vergata Model

The Tor Vergata Model is a physical scattering model based on the Matrix Doubling Algorithm [6,23]. This model is open-source code, which can be obtained from www.disp.uniroma2.it/earth_observation/eraora/urtv/progs/crop1.f (accessed on 9 July 1999). The MDA is an efficient and effective numerical algorithm to solve the Vector Radiative Transfer Equation (VRTE), which is a differential and integral equation for which an analytical solution cannot be found directly. The VRTE is applicable to vegetation that has a large optical thickness. The advantage of the MDA is that it can be used to calculate multiple scattering in a medium, and it has been validated in the simulation of microwave scattering characteristics among forests [18,24], crops [6,25] and snow [26].

**Figure 4:**
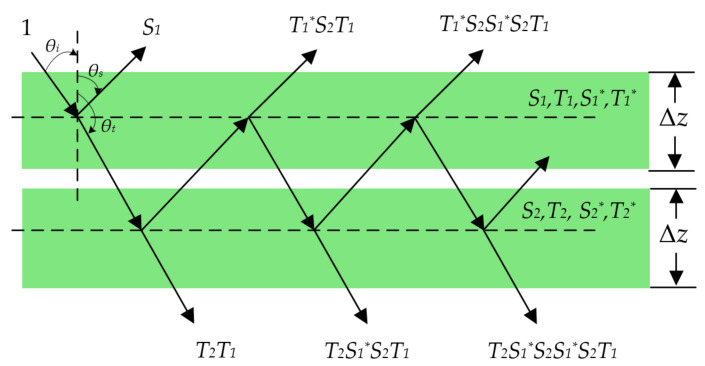
The process of using the Matrix Doubling Algorithm to calculate the multiple scattering of unit incident power between two close thin layers.

In the MDA, the vegetation canopy is horizontally divided into a series of equally thin layers (Figure 4). The thickness of each layer is denoted as Δz, and $P(\theta_{s},\;\theta_{i},\;\phi_{s}-\phi_{i})$ is the scattering phase matrix for each thin layer. The backward scattering matrix S and forward scattering matrix T are then expressed as

(7) $${S(\theta }_{s},\;\theta_{i},\;\phi_{s}-\phi_{i})=U^{-1}P(\theta_{s},\;\theta_{i},\;\phi_{s}-\phi_{i})\Delta z,$$

(8) $$T(\theta_{t},\;\theta_{i},\;\phi_{t}-\phi_{i})=U^{-1}P(\theta_{t},\;\theta_{i},\;\phi_{t}-\phi_{i})\Delta z,$$

where $U^{-1}$ is a diagonal matrix whose elements are the directional cosine of the scattering vector, $\theta_{i}$ and $\phi_{i}$ are the incident and azimuthal angles, $\theta_{s}$ and $\theta_{t}$ denote the backward and forward scattering angles, and $\phi_{s}$ and $\phi_{t}$ denote the backward and forward scattering azimuth angles.

Figure 4 shows the process of using the MDA to calculate the multiple scattering of unit incident power between two close thin layers. The backward and forward scattering matrices of the first layer are $S_{1}$ and $T_{1}$, and those of the second layer are $S_{2}$ and $T_{2}$. The asterisk denotes the backward and forward scattering matrices when the incident direction is reversed. Hence, the backward and forward scattering matrices between the two thin layers with a total thickness of 2Δz can be expressed as

(9) $$S=S_{1}+T_{1}^{*}S_{2}{(1-S_{1}^{*}S_{2})}^{-1}T_{1},$$

(10) $${T=T}_{2}{(1-S_{1}^{*}S_{2})}^{-1}T_{1},$$

(11) $$S^{*}{=S}_{1}{+T}_{1}S_{2}^{*}{(1-S_{1}S_{2}^{*})}^{-1}T_{1}^{*},$$

(12) $$T^{*}{=T}_{2}^{*}\;{(1-S_{1}S_{2}^{*})}^{-1}{\;T}_{1}^{*}.$$

By repeating the above process, we can acquire the backward and forward scattering matrices of the medium layer with a given thickness.

In order to introduce the crown shape to the Tor Vergata Model, we divided the vegetation canopy into a series of thin layers with equal thickness and then established the functional relationship between the envelope equation and the scattering phase matrix $P_{i}{(\theta }_{s}{,\;\theta }_{i},\;\phi_{s}-\phi_{i})$ of each layer. The independent scattering approximation assumes that the scattered fields of vegetation components are independent of each other [27]; that is, the coherent effects among vegetation components can be neglected. As a result, the scattering phase matrix can be obtained as

(13) $${P(\theta }_{s}{,\;\theta }_{i},\;\phi_{s}-\phi_{i})=\overset{n}{\underset{j=0}{\sum }}N_{j}{(h)\;P}_{i}{(\theta }_{s}{,\;\theta }_{i},\;\phi_{s}-\phi_{i}),$$

where *n* is the number of scatterer types within the canopy, and $N_{j}\left(h\right)$ is the number of the *j*-th kind of scatterer. $N_{j}\left(h\right)$ is defined as

(14) $$N_{j}(h)=\int_{0}^{2\pi }d\phi \int_{h}^{h+\Delta z}dz\int_{0}^{F(\phi ,z)}\mu_{j}\rho (\phi ,\;h)\;d\rho ,$$

where $\mu_{j}$ (m^−3^) is the volume density of the *j*-th kind of scatterer.

Therefore, the crown shape ρ(ϕ, h) is related to the backscattering coefficients by $N_{j}\left(h\right)$ and $P_{i}{(\theta }_{s}{,\;\theta }_{i},\;\phi_{s}-\phi_{i})$. For an ideal crown shape, which is obtained by rotating a function curve F(h) around the *h*-axis, the crown envelope equation is ρ(h)=F(h). Assuming that $\mu_{j}(\rho ,\;\phi ,\;h)$ is constant within the canopy, it is substituted into Equation (14), which can then be further simplified to

(15) $$N_{j}{(h)=\pi \mu }_{j}\int_{h}^{h+\Delta z}F^{2}(h)\;dz.$$

In this study, the Modified Tor Vergata Model was used to simulate the backscattering coefficients of the three ideal vegetation canopies. Without considering the crown shape, most of the traditional scattering models assume a constant vegetation volume fraction in the vertical dimension, which is equivalent to treating the crown as a cylinder, so a cylindrical crown shape was also included as a reference.

As this is preliminary research, we forgo the realistic description of real vegetation canopies, and more comprehensive simulation experiments will be conducted in future work. Two kinds of ideal vegetation canopy, A and B, were considered in the current simulation. The vegetation components of canopy A were broad leaves and stalks, and the vegetation components of canopy B were needle leaves and branches. The parameters of canopy A (Table 1) were obtained from ground experiments on rice, which were carried out in Jiangsu Province (33°6′59″ N, 118°58′16″ E) [28,29]. As the stalk extended from the top of the vegetation canopy to the bottom, the stalk length was shortened to fill the canopy space as a small vegetation component. The canopy height also lost its practical significance in the rice field. The parameters of canopy B (Table 2) were obtained from a ground experiment on forest, which was carried out in Guizhou Province (26°53′45″, 106°45′30″) [30]. The branch parameters were obtained from branches in the second level, and the length was also shortened.

The scatterer permittivity was computed through the semiempirical formula given by Ulaby and El-Rayes [31], which needs the moisture content of the scatterer as an input. The computational complex dielectric constants of canopies A and B are calculated as

(16) $$\varepsilon_{A}=35.614-11.1356\;i,$$

(17) $$\varepsilon_{B}=19.2601-6.2882\;i,$$

where *i* denotes $\sqrt{-1}$.

Without loss of generality, the volume density of all vegetation components was empirically fixed at 8 × 10^−5^ cm^−3^ in the simulation. The vegetation canopy scene was assumed to be irradiated by a homogeneous plane wave. The scattering phase matrix of disc leaves, needle leaves and stalks (or branches) were calculated using physical optics approximation [32], Rayleigh–Gans approximation [33] and infinite length approximation [34], respectively. We simulated the variation in the canopy backscattering coefficients at different canopy heights for VV, HH, VH and HV polarizations of L (1.2 GHz), C (5.3 GHz) and X (9.6 GHz) bands. The simulation results are shown in Section 3.1.

### 2.3. Vegetation Canopy Modeling and Simulation with FEKO

As described in Section 2.2, the backscattering coefficients of four ideal canopies with different crown shapes were simulated using the Modified Tor Vergata Model so as to quantitatively analyze the effects of crown shape on microwave backscattering coefficients. However, ideal vegetation canopies that have different crown shapes but identical canopy parameters are rare in nature, making it difficult to carry out experimental measurements only for the vegetation canopy. Therefore, numerical computation was used to simulate the backscattering coefficients of the four ideal canopies with different crown shapes. FEKO is comprehensive computational electromagnetic software that is used to calculate 3D electromagnetic fields with numerical methods, and the simulation results have high reliability and have been validated in many applications [35,36,37,38,39,40].

FEKO includes multiple frequency- and time-domain solution methods, which can be grouped into full-wave solution methods and asymptotic solution methods [20]. Full-wave solution methods include Method of the Moment (MOM), Multilevel Fast Multipole Method (MLFMM), Finite Element Method (FEM) and Finite-Difference Time-Domain (FDTD). Asymptotic solution methods include Physical Optics (PO), Large Element Physical Optics (LE-PO), Ray-Launching Geometrical Optics (RL-GO) and Uniform Theory of Diffraction (UTD), among others. The difference between full-wave solutions and asymptotic solutions is whether the solutions make certain assumptions or not when solving Maxwell’s equations, and full-wave solutions are considered more accurate when not making any assumptions. Among the numerical methods used, the MOM is the default solver in FEKO, and the MLFMM is an alternative formulation of the technique and applies to much larger structures than the MOM (in terms of the wavelength), but its calculations consume less memory and time under the same circumstances. The maximum mesh number of the 3D vegetation canopies was 2.15 million in this analysis; thus, considering the amount of computation, the MLFMM was a better choice to compute the backscattering coefficients of vegetation canopies. This section establishes 3D geometrical vegetation canopies and describes simulations with FEKO and its script language, LUA.

**Figure 5:**
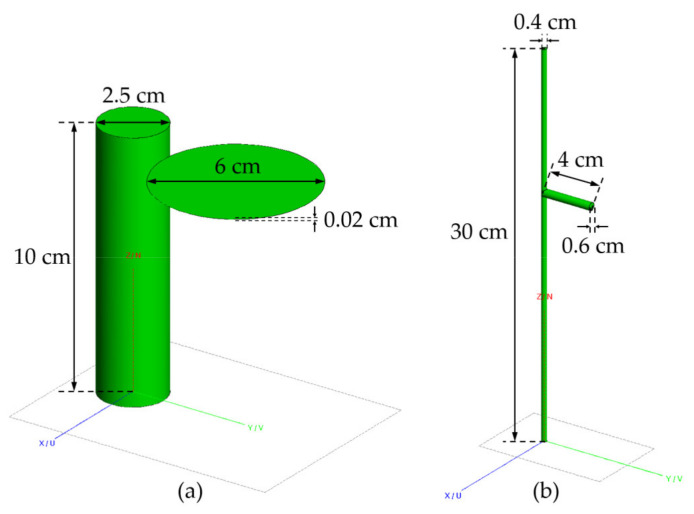
Two different structures of vegetation canopy components: (**a**) the component structure of canopy A; (**b**) the component structure of canopy B.

Different geometries were used to model vegetation components of canopies A and B. For canopy A, discs with small thickness and cylinders with finite length were used to model leaves and stalks (Figure 5). For canopy B, leaves and branches were both simulated with finite-length cylinders.

Similarly, to ensure that the crown shape was the only variable in modeling 3D geometrical vegetation canopies, a 3D canopy model was built based on the same assumptions, except that the 3D vegetation canopies were divided into layers in the *h* direction for model simplification, and every leaf in 3D vegetation canopies was randomly attached to the surface of one stalk (or branch).

The arrangement of the canopy components differs among the four vegetation canopies with the same canopy height. For example, the cone canopy is divided into $N_{l}$ layers from the vertex to the bottom surface, and $N_{l}=\lfloor \frac{H}{l}\rfloor$, where *l* denotes the length of the stalk or branch, ⌊ ⌋ denotes the round-down function, and the number of vegetation components is proportional to the corresponding volume. In the *i*-th layer, the vegetation components are uniformly distributed, and the exact position of every component is decided by collision detection [41]. Among the layers, the vegetation components do not intersect with each other.

The number of vegetation components in each layer is determined as follows. For the cylinder canopy, the vegetation component number of the *i*-th layer is $N_{i}$, and $N_{i}=\lceil \frac{\rho_{v}V_{h}}{N_{l}}\rceil$, where $V_{h}$ denotes the volume of the cylinder canopy with height *h*, with h=0.8, 0.9, …, 2.9, 3.0, and ⌈ ⌉ denotes the round-up function. For the cone, inverted cone and ellipsoid canopies, $N_{i}=\rho_{v}V_{i}$, where $V_{i}$ denotes the volume of the *i*-th layer, and ${i=1,\;2,\;\ldots ,\;N}_{l}-{1,\;N}_{l}$. Specifically, every layer of the cylinder canopy has an equal volume $V_{i}$; in other words, the number of vegetation components in each layer is the same, which is $\left\lceil \frac{\rho_{v}V_{h}}{N_{l}}\right\rceil$ or $\rho_{v}V_{i}$.

The *i*-th layer volume of the cone canopy gradually increases from the vertex to the bottom surface, and the volume of the *i*-th layer can be calculated by

(18) $$V_{i}{=V}_{h}-\frac{1}{3}{\pi (r}_{i}-{\;l\tan\alpha )}^{2}\left(H-\mathrm{il}\right)-V_{t},$$

where $V_{h}$ denotes the volume of the cone canopy with height *h*, with h=0.8, 0.9, …, 2.9, 3.0, and $V_{t}$ is a temp variable with an initial value of 0. $r_{i}$ and $V_{t}$ can be obtained by

(19) $$\left\{\begin{array}{l} r_{i}=\left(H-\left(i-1\right)l\right)\tan\alpha \\ V_{t}{=V}_{i}{+V}_{t} \end{array}\right..$$

The inverted cone canopy is quite the opposite of the cone canopy. The *i*-th layer volume of the inverted cone canopy gradually decreases from the top surface to the bottom vertex. $V_{i}$, $r_{i}$ and $V_{t}$ can be obtained by

(20) $$\left\{\begin{array}{l} V_{i}=\frac{1}{3}{\mathrm{il}\pi r}_{i}^{2}-V_{t}\\ r_{i}=\mathrm{il}\tan\alpha \\ V_{t}{=V}_{i}{+V}_{t} \end{array}\right..$$

For the ellipsoid canopy, the *i*-th layer volume first increases and then decreases from the top vertex to the bottom vertex, and $r_{i}$ can be grouped into two parts:

(21) $$\mathrm{when}\;i>\lceil \frac{N_{l}}{2}\rceil ,\;r_{i}=\sqrt{2l\left(i-1\right)\left(H-\left(i-1\right)l\right)}\tan\alpha ,$$

(22) $$\mathrm{when}\;i\leq \lceil \frac{N_{l}}{2}\rceil ,\;r_{i}=\sqrt{2l\left(i-1\right)\left(H-\mathrm{il}\right)}\tan\alpha ,$$

and the volume of the *i*-th layer can be written as

(23) $$\left\{\begin{array}{l} V_{i}=\pi \left(-\frac{2}{3}{\left(\frac{i}{10}\right)}^{3}+{\left(\frac{i}{10}\right)}^{2}H\right)-V_{t}\\ V_{t}{=V}_{i}{+V}_{t} \end{array}\right..$$

Table 3 shows the number of layers of the four canopies with different crown shapes and the number of vegetation components in each layer.

**Figure 6:**
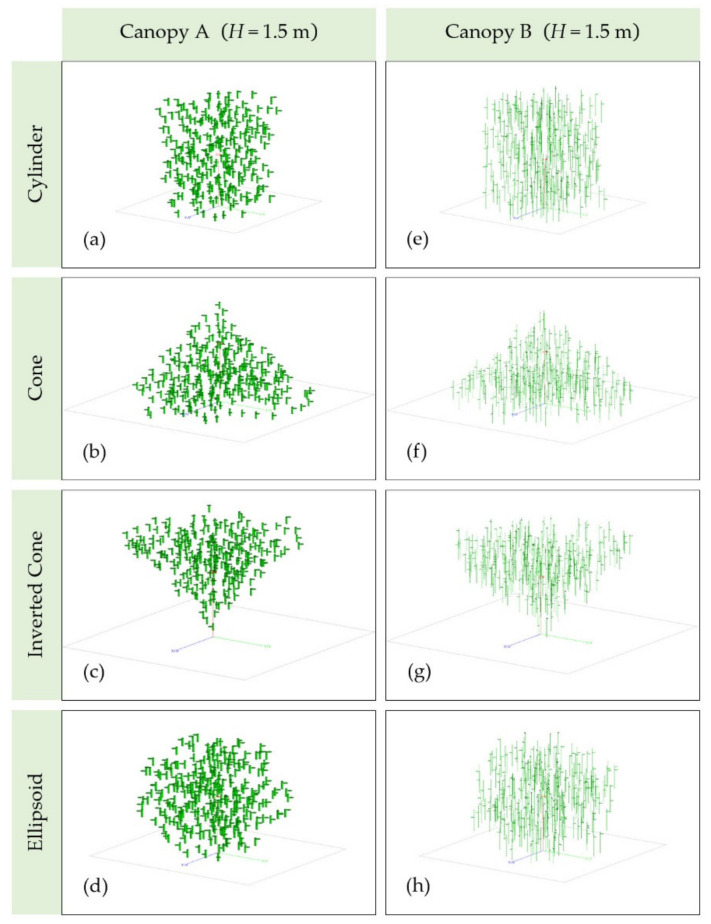
The four 3D geometrical vegetation canopies with different crown shapes when *H* = 1.5 m. In (**a**–**d**), the parameters from Table 1 were used as model inputs; in (**e**–**h**), the parameters from Table 2 were used as model inputs.

After determining the number of layers and vegetation components, the four 3D geometrical vegetation canopies with different crown shapes were established using the LUA script in FEKO. Figure 6 shows the corresponding 3D geometrical vegetation canopies with two sets of parameters as model inputs when *H* = 1.5 m.

Simulating backscattering coefficients of vegetation canopies in FEKO consists of the following steps, and the simulation results are shown in Section 3.2.

1. Modeling 3D geometrical vegetation canopies, as shown in Figure 6.
2. Setting parameters, which include the media properties (such as structure and permittivity), frequencies, wave sources, polarizations and solution requests. The computational complex dielectric constants of canopies A and B are shown in Equations (16) and (17). The frequency was set at 5.3 GHz, the incident angle was 43°, the azimuthal angles ranged from 0° to 360° with an interval of 45°, and the wave source was set as a plane wave at VV and VH polarizations.
3. Creating mesh, which is related to the solution methods, dielectric properties, frequencies, geometry curvature, etc. FEKO provides three mesh options to automatically determine the appropriate mesh size for the model: coarse, standard or fine mesh can be selected based on the required accuracy and the available computational resources. In addition, a custom and local mesh size can also be set without applying the automatic meshing algorithm. In this study, the coarse mesh was applied to the vegetation canopies for the sake of computation efficiency.
4. Choosing the solution method, which depends mainly on the model mesh size and computational efficiency. Because the maximum mesh of the 3D vegetation canopy models is more than two million, the MLFMM was chosen in this study, and the box size in wavelength was set at 0.21 to improve the convergence.
5. Running the solver and analyzing results. The computation was conducted on a server with four Intel(R) Xeon(R) Gold 6252 CPUs. Every CPU has 24 cores in the server; the CPU clock speed is 2.10 GHz, and the total memory is 2 TB. For the maximum mesh size, it takes about six hours to compute a single result (for a single frequency, single incident angle and single azimuthal angle).

## 3. Results and Discussion

### 3.1. MTVM Simulation Results

**Figure 7:**
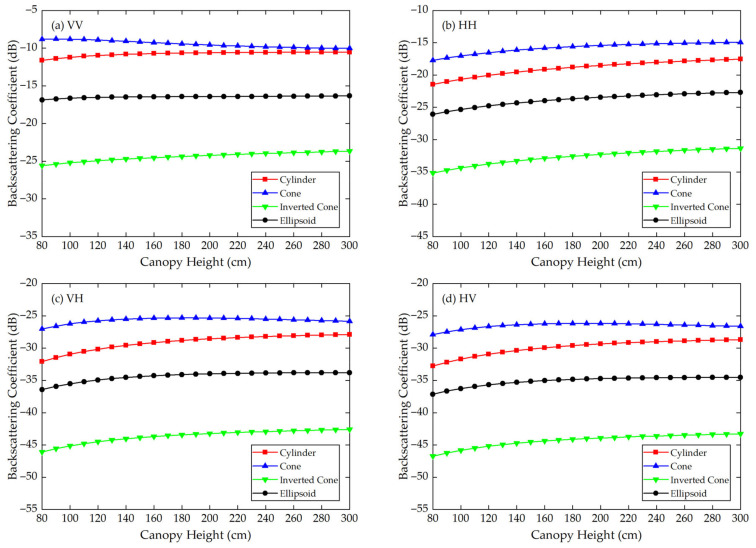
The MTVM simulation results of the backscattering coefficients for different crown shapes in L band (1.2 GHz) at (**a**) VV, (**b**) HH, (**c**) VH and (**d**) HV polarizations. The model input parameters are from Table 1 (canopy A).

**Figure 8:**
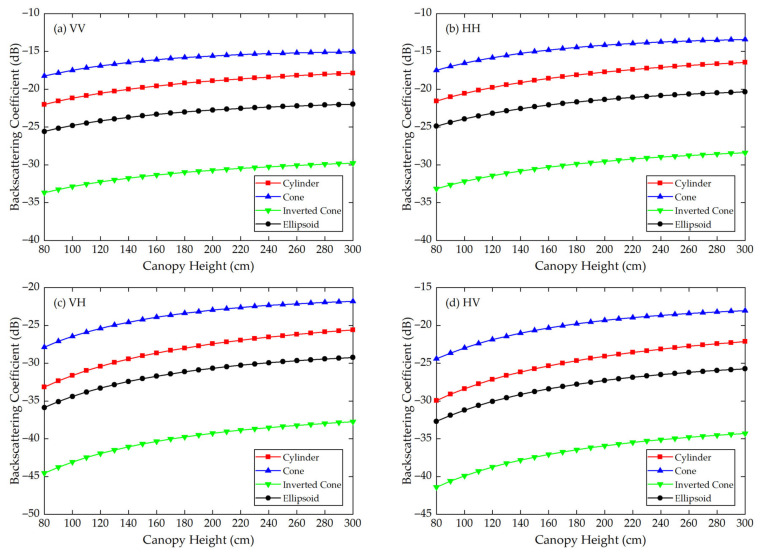
The MTVM simulation results of the backscattering coefficients for different crown shapes in C band (5.3 GHz) at (**a**) VV, (**b**) HH, (**c**) VH and (**d**) HV polarizations. The model input parameters are from Table 1 (canopy A).

**Figure 9:**
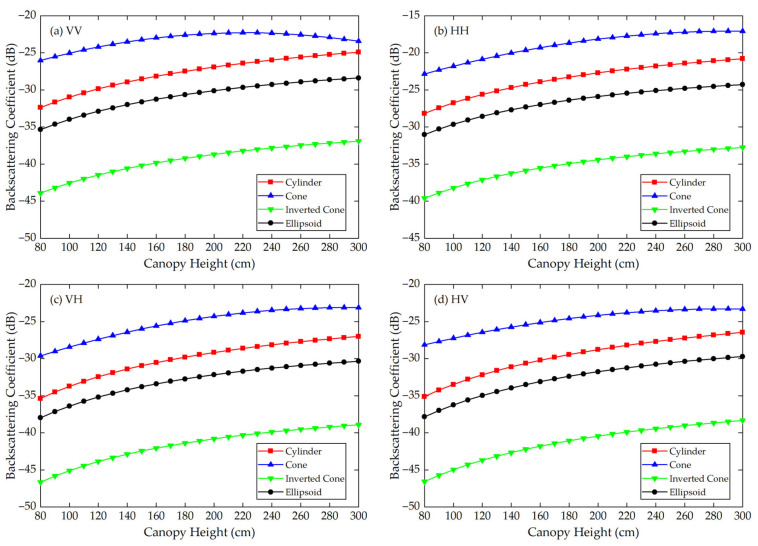
The MTVM simulation results of the backscattering coefficients for different crown shapes in X band (9.6 GHz) at (**a**) VV, (**b**) HH, (**c**) VH and (**d**) HV polarizations. The model input parameters are from Table 1 (canopy A).

**Figure 10:**
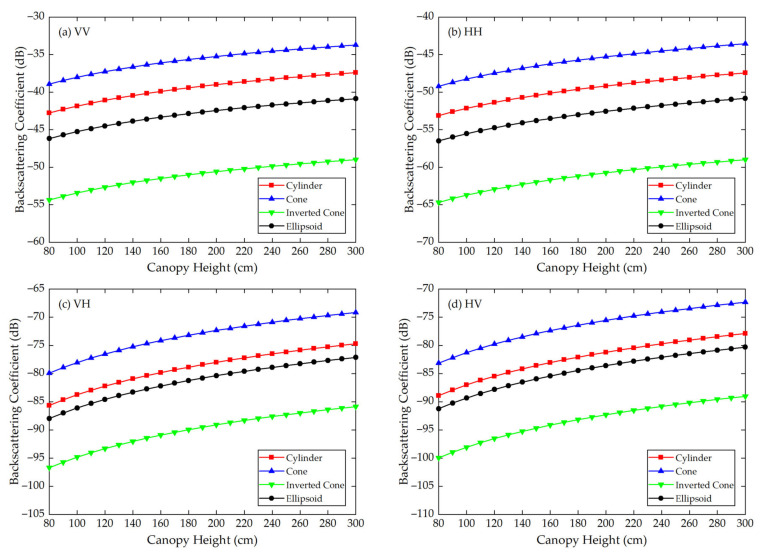
The MTVM simulation results of the backscattering coefficients for different crown shapes in L band (1.2 GHz) at (**a**) VV, (**b**) HH, (**c**) VH and (**d**) HV polarizations. The model input parameters are from Table 2 (canopy B).

**Figure 11:**
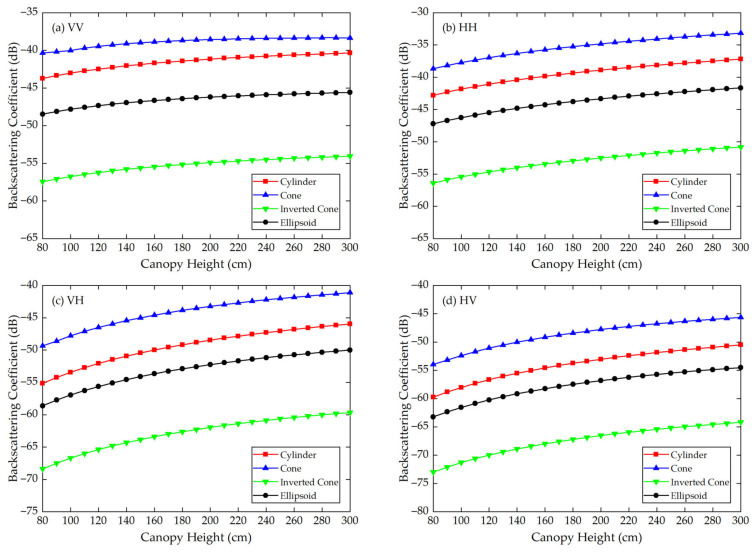
The MTVM simulation results of the backscattering coefficients for different crown shapes in C band (5.3 GHz) at (**a**) VV, (**b**) HH, (**c**) VH and (**d**) HV polarizations. The model input parameters are from Table 2 (canopy B).

**Figure 12:**
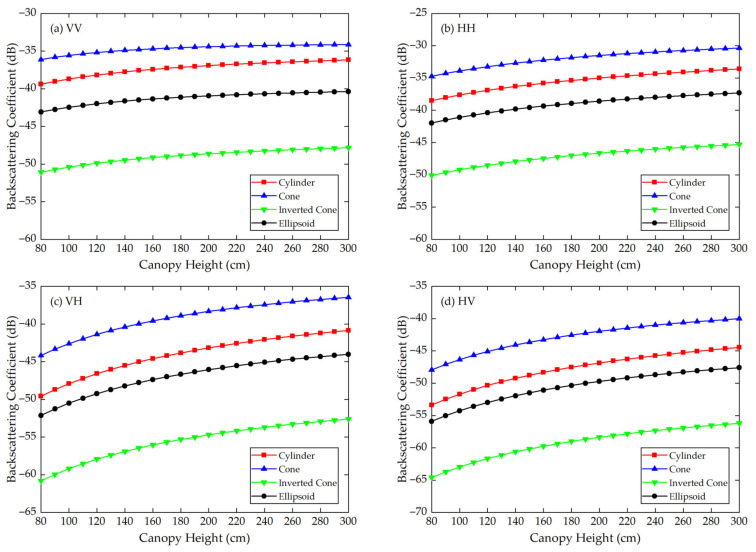
The MTVM simulation results of the backscattering coefficients for different crown shapes in X band (9.6 GHz) at (**a**) VV, (**b**) HH, (**c**) VH and (**d**) HV polarizations. The model input parameters are from Table 2 (canopy B).

Using the Modified Tor Vergata Model, the simulation results for the four vegetation canopies with different crown shapes are shown in Figure 7, Figure 8, Figure 9, Figure 10, Figure 11 and Figure 12. Figure 7, Figure 8 and Figure 9 illustrate the results at VV, HH, VH and HV polarizations in L (1.2 GHz), C (5.3 GHz) and X (9.6 GHz) bands with the parameters of canopy A as inputs; Figure 10, Figure 11 and Figure 12 show analogous results but with the parameters of canopy B as inputs. The four vegetation canopies have vegetation components with the same volume density ($\rho_{v}=$ 8 × 10^−5^ cm^−3^), and the crown heights of the four canopies remain the same (*H* = 80–300 cm).

For canopies A and B, at VV, HH, HV and VH polarizations in L, C and X bands, Figure 7, Figure 8, Figure 9, Figure 10, Figure 11 and Figure 12 all show that the backscattering coefficients of vegetation canopies with different crown shapes increase gradually as the canopy height increases from 80 cm to 300 cm, except for the cone canopy at VV, VH and HV polarizations in the L band and at VV polarization in the X band, for which the MTVM simulation results slightly decrease with the increase in crown height.

The mean absolute difference (MAD) $\bar{\mu_{a}}$ and the mean relative difference (MRD) $\bar{\mu_{r}}$ were used to evaluate the differences between the backscattering coefficients for the three crown shapes studied and the reference cylinder crown shape. The results are presented in Table 4 and Table 5. For two series of numbers $\left\{x_{n}\right\}$ and $\left\{x_{n}^{\prime }\right\}$, expressed in dB, $\bar{\mu_{a}}$ and $\bar{\mu_{r}}$ are defined as

(24) $$\bar{\mu_{a}}=\frac{1}{n}\overset{n}{\underset{i=1}{\sum }}\left|x_{i}-x_{i}^{\prime }\right|,$$

(25) $$\bar{\mu_{r}}=\frac{1}{n}\overset{n}{\underset{i=1}{\sum }}\left|\frac{x_{i}-x_{i}^{\prime }}{x_{i}}\right|,$$

The units of $\bar{\mu_{a}}$ and $\bar{\mu_{r}}$ are dB and percent, which we do not repeatedly mention in the following content. In Table 4 and Table 5, the upper row is $\bar{\mu_{a}}$ and the lower row is $\bar{\mu_{r}}$ for every polarization.

For canopy A, the largest $\bar{\mu_{a}}$ values between the three crown shapes studied and the reference cylinder crown shape are 14.55 dB, 11.82 dB and 11.76 dB, and they occur at VH (L band), HV (C band) and VV (X band) polarizations, respectively. The smallest $\bar{\mu_{a}}$ values occur at VV (L band), HV (C band) and VH/HV (X band) polarizations, and the corresponding values are 1.32 dB, 3.17 dB and 2.97 dB. Moreover, the largest $\bar{\mu_{a}}$ occurs between the inverted cone and reference cylinder canopies, and the smallest occurs between the cone and reference cylinder canopies for the L band and between the ellipsoid and cylinder canopies for C and X bands.

The largest $\bar{\mu_{r}}$ values for canopy A are 126.68%, 64.87% and 50.26% and occur between the inverted cone and cylinder canopies at VV (L band) and HH (C and X bands) polarizations. The smallest values occur between cone and cylinder canopies at HV (L band) polarization with a value of 11.17% and between the ellipsoid and cylinder canopies at VH (C and X bands) polarization with values of 11.39% and 9.99%.

Analogously, for canopy B, the largest $\bar{\mu_{a}}$ values between the three crown shapes studied and the reference cylinder crown shape are 11.60 dB, 13.74 dB and 11.70 dB, which occur between the inverted cone and cylinder canopies at VV (L, C and X bands) polarization. The smallest $\bar{\mu_{a}}$ values occur between the ellipsoid and cylinder canopies at HV (L band) polarization with a value of 2.37 dB and between the cone and cylinder canopies at VV (C and X bands) polarization with values of 2.63 dB and 2.59 dB.

The largest $\bar{\mu_{r}}$ values for canopy B are 29.41%, 34.61% and 32.90% and occur between the inverted cone and cylinder canopies at VV (L band) and HH (C and X bands) polarizations. The smallest values occur between the ellipsoid and cylinder canopies at HV (L and X bands) polarization with values of 2.88% and 5.94% and between the cone and cylinder canopies at VV (C band) polarization with a value of 6.31%.

For the given parameters, the maximum mean absolute difference in backscattering coefficients between different crown shapes is 14.55 dB (for canopy A at VH polarization in L band), and the maximum mean relative deviation between the studied canopy and the reference cylinder canopy is 126.68% (for canopy A at VV polarization in L band) (Table 4). Taking Figure 9a as an example, the mean absolute differences between the cone, inverted cone and ellipsoid canopies and the reference cylinder canopy are 4.40 dB, 11.76 dB and 3.19 dB, and the absolute differences between values for the cone and inverted cone are as high as about 18 dB. Therefore, it can be concluded that the crown shape has a non-negligible influence on microwave backscattering coefficients of the vegetation canopy.

The ranking of backscattering coefficients is $\sigma_{\mathrm{cone}}^{0}>\sigma_{\mathrm{cylinder}}^{0}>\sigma_{\mathrm{ellipsoid}}^{0}>\sigma_{\mathrm{inv}-\mathrm{cone}}^{0}$, which is approximately the same ranking order as that of the volume fraction of the lower half of the vegetation canopy, for which the fractions are cone (75%) > cylinder (50%) = ellipsoid (50%) > inverted cone (25%). This correspondence can possibly be attributed to the attenuation effects of the upper canopy components; in other words, a lower volume fraction produces less attenuation, increasing the backscattering coefficients of the lower part of the canopy. However, a quantitative explanation of this result requires further analysis.

Moreover, we can see in Figure 7, Figure 8, Figure 9, Figure 10, Figure 11 and Figure 12 that, for a given crown shape, the backscattering coefficients and their relative differences for A are both greater than those for B in the same band and polarization. This indicates that the crown shape effect acts synergistically with the effects of vegetation component parameters, which mainly include the geometrical and physical parameters of the vegetation components.

In addition, we also note that a great difference exists between the cone and inverted cone canopies. A set of curves is used to illustrate the gradualness of the continuous change from the cone canopy to the inverted cone canopy and its influence on the backscattering coefficients of the vegetation canopy. For the four crown shapes, the horizontal profiles at a certain height are all circles whose areas can be expressed with a quadratic curve as ${\pi \rho }^{2}\left(h\right)$, so the parabolic curve is the best choice to represent the continuous change in crown shapes. The parabolic vegetation canopy can be defined as

(26) $$\rho^{2}\left(h\right){=\mathrm{ah}}^{2}+\mathrm{bh}+c.$$

Likewise, to ensure that the crown shape is the unique variable of the vegetation canopy, assuming that all other canopy parameters are the same, the volume of the three vegetation canopies in Figure 3 ought to remain the same as that of the reference cylinder canopy. It is defined as

(27) $$\pi \int_{0}^{H}\rho^{2}(h)\;\mathrm{dh}=\frac{1}{3}{\pi R}^{2}H,$$

By integrating Equation (27), we obtain

(28) $$\frac{a}{3}+\frac{b}{2H}+\frac{c}{H^{2}}=\frac{\tan^{2}\alpha }{3}.$$

As a result, the parabolic vegetation canopies should meet the requirements in Equation (28), which constrains the geometries of the crown shapes. The constraint conditions are as follows

(29) $$\left\{\begin{array}{l} \frac{a}{3}+\frac{b}{2H}+\frac{c}{H^{2}}=\frac{\tan^{2}\alpha }{3}\\ {\mathrm{ah}}^{2}+\mathrm{bh}+c\geq 0,\;h\in \left[0,\;H\right] \end{array}\right..$$

**Figure 13:**
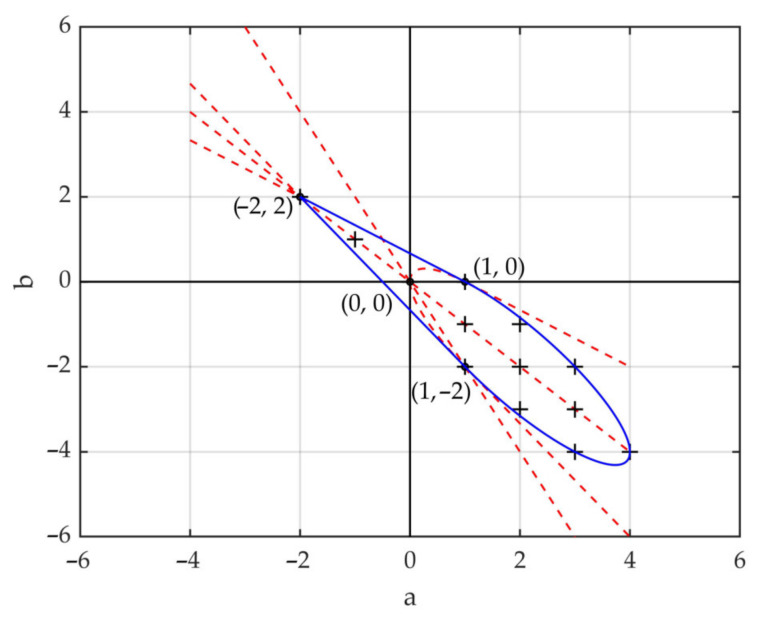
The feasible region of parabola factors a and b. The blue line represents the feasible region, the cross symbols represent the integer feasible solutions, and the bold dots with coordinates denote the solutions of a and b, which correspond to the four crown shapes.

In particular, when *H* = 1 m and α=45°, the feasible region of parabola factors a and b is confined by the blue line in Figure 13. In the figure, the blue line represents the feasible region boundary of a and b, the cross symbols represent the integer feasible solutions, and the four bold dots with coordinates correspond to the four crown shapes, respectively. The crown shapes and the corresponding values of factors a, b and c are as follows.

- Cylinder: a = 0, b = 0, c = 1/3;
- Cone: a = 1, b = −2, c = 1;
- Inverted Cone: a = 1, b = 0, c = 0;
- Ellipsoid: a = −2, b = 2, c = 0.

**Figure 14:**
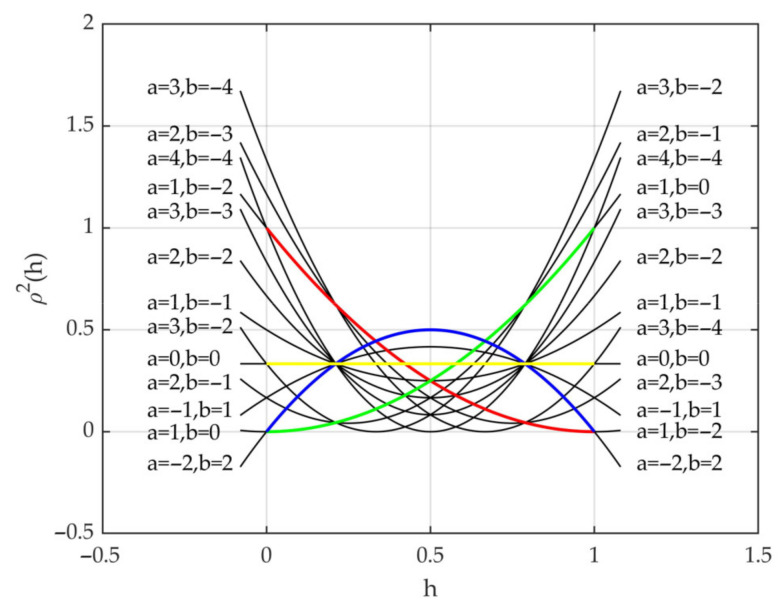
The squares of the parabola equations with different integer feasible solutions, which correspond to the cross symbols in Figure 13. The yellow, green, red and blue represent the square of the cylinder, cone, inverted cone and ellipsoid equations, respectively, and correspond to the bold dots in Figure 13.

Figure 14 shows the squares of the parabola equations with different integer feasible solutions, which correspond to the cross symbols in Figure 13.

**Figure 15:**
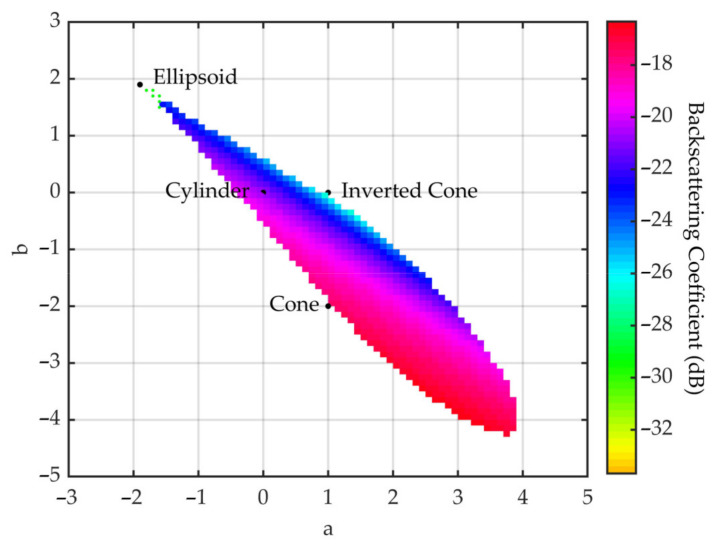
Backscattering coefficient simulation results of different crown shapes with discrete a and b in the feasible region; the bold dots correspond to the four crown shapes.

Furthermore, in the feasible region, we chose parabola factors a and b with 0.1 as the interval and calculated factor c with Equation (28). Then, the backscattering coefficients of the corresponding vegetation canopies were simulated at VV polarization in C band, with the parameters of canopy A as the Modified Tor Vergata Model input, and the results are presented in Figure 15. In Figure 15, the bold dots represent the four crown shapes and correspond to the points in Figure 13, and different colors correspond to different backscattering coefficients. From Figure 15, we can conclude that the variation in the backscattering coefficients maintains good continuity over different crown shapes.

**Figure 16:**
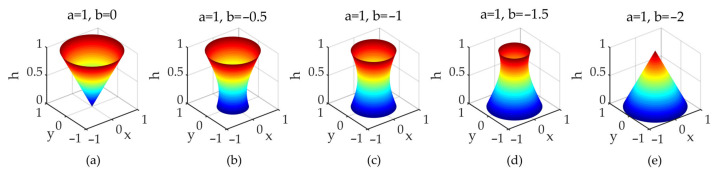
Rendering graphs of five transition crown shapes from the inverted cone to the cone. The parabola factors are (**a**) a = 1, b = 0; (**b**) a = 1, b = −0.5; (**c**) a = 1, b = −1; (**d**) a = 1, b = −1.5; and (**e**) a = 1, b = −2.

Specifically, three transition points were chosen between the cone and inverted cone to form a point set, written as {(a, b): (1, 0), (1, −0.5), (1, −1), (1, −1.5), (1, −2)}. Figure 16 shows successive transformations of the crown shapes corresponding to the point set from (a) to (e); the corresponding backscattering coefficients (as shown in Figure 15) of the vegetation canopies decrease gradually, which is a good indication that the continuous transformation of the crown shapes gives rise to the variation in backscattering coefficients of the corresponding vegetation canopies.

### 3.2. FEKO Simulation Results

**Figure 17:**
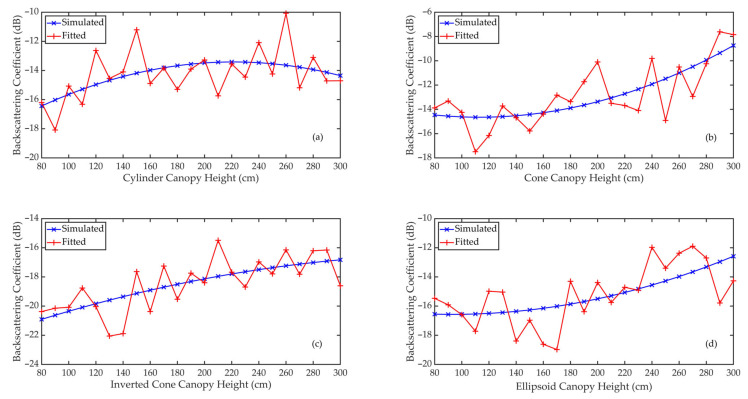
The simulated and fitted backscattering coefficients of vegetation canopies when using canopy A’s parameters as inputs at different canopy heights.

**Figure 18:**
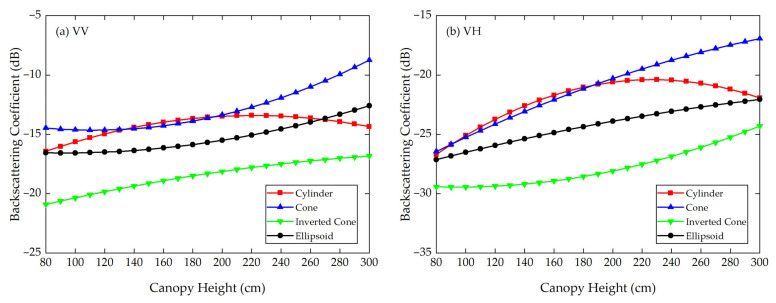
The averaged FEKO simulation results of the backscattering coefficients for different crown shapes at VV and VH polarizations of C band (5.3 GHz) when using canopy A’s parameters as inputs. (**a**) VV polarization; (**b**) VH polarization.

**Figure 19:**
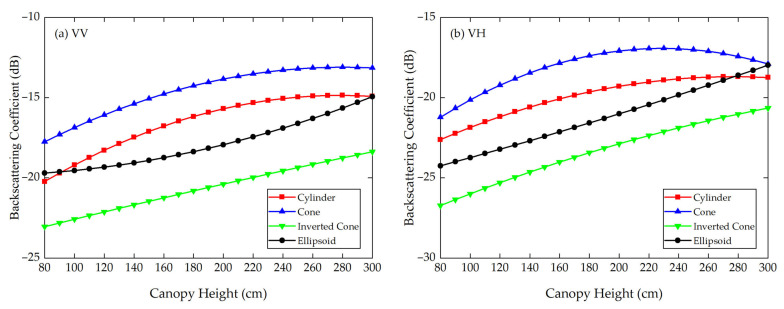
The averaged FEKO simulation results of the backscattering coefficients for different crown shapes at VV and VH polarizations of C band (5.3 GHz) when using canopy B’s parameters as inputs. (**a**) VV polarization; (**b**) VH polarization.

Considering the long computation time and high memory requirements of FEKO, we only simulated the variation in the canopy backscattering coefficients at different canopy heights for the VV and VH polarizations in the C band (5.3 GHz). The simulation results are shown in Figure 17, Figure 18 and Figure 19.

In Figure 17, the red lines represent the averaged simulation results of eight azimuthal angles in FEKO when using canopy A’s parameters as inputs, and the blue lines denote the quadratic polynomial fitting results of the simulation results, which are in good agreement with the averaged simulation results; (a), (b), (c) and (d) correspond to the simulation results of the cylinder, cone, inverted cone and ellipsoid canopies at VV polarization of the C band. The processes of fitting at VH polarization for canopy A and at VV and VH polarizations for canopy B are the same as in Figure 17, so they are not repeated here. For different canopy heights, Figure 18 and Figure 19 show the fitted FEKO results for canopies A and B with different crown shapes at VV and VH polarizations of the C band. On the whole, the fitted backscattering coefficients increase gradually as the canopy height increases from 80 cm to 300 cm.

In the same way, the mean absolute difference $\bar{\mu_{a}}$ and the mean relative difference $\bar{\mu_{r}}$ were used to evaluate the differences between the simulated backscattering coefficients for the three crown shapes studied and the reference cylinder crown shape. The results are presented in Table 6, in which the upper row is $\bar{\mu_{a}}$ and the lower row is $\bar{\mu_{r}}$ for a certain polarization.

For canopies A and B in Table 6, the largest MAD and MRD both occur between the inverted cone and cylinder canopies. Specifically, for canopy A, the largest MAD is 5.76 dB at VH polarization, and the largest MRD is as high as 31.65% at VV polarization. The smallest MAD is 1.84 dB between the ellipsoid and cylinder canopies at VV polarization, and the smallest MRD is 10.48% between the cone and cylinder canopies at VH polarization. Accordingly, for canopy B, the maximum MAD and MRD are 4.17 dB and 26.56%, both occurring between the inverted cone and cylinder canopies, and the minimum MAD and MRD are 2.08 dB and 10.77%, both occurring between the ellipsoid and cylinder canopies.

Taking canopy A as an example, for VV polarization at the C band, we can see from Table 6 that different crown shapes produce a significant MAD (4.31 dB) in the backscattering coefficients, with the MRD reaching as high as 31.65% (VV polarization). At VH polarization, the MAD and MRD reach 5.76 dB and 27.22%. Furthermore, the absolute difference between the values for the cone and inverted cone at VH polarization is as high as 12 dB when the crown height is 220 cm. Hence, it can also be concluded that the crown shape has a non-negligible influence on microwave backscattering coefficients of the vegetation canopy at the given frequency and polarizations.

Overall, for FEKO simulation results at VV and VH polarizations of the C band, the ranking of the backscattering coefficients of the four vegetation canopies is $\sigma_{\mathrm{cone}}^{0}>\sigma_{\mathrm{cylinder}}^{0}>\sigma_{\mathrm{ellipsoid}}^{0}>\sigma_{\mathrm{inv}-\mathrm{cone}}^{0}$, which is approximately the same as the ranking of the volume fraction of the lower half of the vegetation canopy, for which the fractions are cone (75%) > cylinder (50%) = ellipsoid (50%) > inverted cone (25%). To be specific, there are some differences between the results of MTVM and FEKO. As shown in Figure 18a for canopy A, the backscattering coefficients of the cylinder canopy are a little higher than those of the cone canopy when the canopy height is between 140 cm and 190 cm, and they are smaller than those of the ellipsoid canopy after 270 cm. In Figure 18b, the backscattering coefficients of the cylinder canopy are slightly higher than those of the ellipsoid canopy at canopy heights between 100 cm and 180 cm.

Furthermore, it is clear that the backscattering coefficients and their relative differences for canopy A are greater than those for canopy B at VV polarization of the C band, but they are lower than those for canopy B at VH polarization of the C band. This is probably because of the difference in the geometrical and physical parameters of the vegetation components.

### 3.3. Comparative Analysis

In this section, the mean absolute difference $\bar{\mu_{a}}$ and the mean relative difference $\bar{\mu_{r}}$ are used to evaluate differences in the MTVM and FEKO simulation results between canopies A and B. The MADs and MRDs between canopies A and B for the same crown shapes are shown in Table 7 and Table 8, which show the MTVM and FEKO simulation results, respectively. In Table 7 and Table 8, $\bar{\mu_{a}}$ denotes the mean absolute difference between canopies A and B for the same crown shape, and $\bar{\mu_{rA}}$ and $\bar{\mu_{rB}}$ denote the mean relative differences for canopies A and B. The units remain the same as previously described.

For the MTVM simulation results at VV polarization of the C band, the maximum MAD between canopies A and B is 24.12 dB for the inverted cone canopy, and the minimum MAD is 22.18 dB for the cylinder canopy. The maximum MRD for canopy A is as high as 143.28% for the cone canopy. The MRDs of canopy A are greater than those of canopy B, but the magnitudes remain the same. The results at VH polarization follow similar rules.

For the FEKO simulation results at VV polarization of the C band, the maximum MAD between canopies A and B is 3.09 dB for the ellipsoid canopy, and the minimum MAD is 2.47 dB for the inverted cone canopy, which does not considerably differ from the others. The MRDs for canopy A are larger than those for canopy B, and the maximum MRD is 22.83% for the cone canopy at VV polarization. The FEKO results at VH polarization are bound by similar rules, but the MRDs for canopy B are larger than those for canopy A at VH polarization.

We can also notice that the MADs and MRDs greatly differ between the MTVM and FEKO simulation results. By contrasting Figure 8 and Figure 18 and Figure 11 and Figure 19, the results show that the FEKO simulation results are generally higher than the MTVM simulation results at VV and VH polarizations of the C band (5.3 GHz), which may be due to different unreasonable assumptions or the parameter settings of the two simulations. The actual reasons need to be further explored by analytical simulation and experimental measurement, which will be the focus of our future work.

## 4. Conclusions

The main aim of this paper is to develop a method to quantitatively describe the effects of crown shape on the microwave backscattering coefficients of the vegetation canopy. With the use of the Modified Tor Vergata Model and electromagnetic numerical method, the variations in backscattering coefficients from vegetation canopies with different crown shapes were simulated. The main conclusions can be summarized as follows.

- Using the Modified Tor Vergata Model, the backscattering coefficients of the cylinder, cone, inverted cone and ellipsoid canopies with different crown heights (*H* = 80–300 cm) were simulated for canopies A and B at VV, HH, VH and HV polarizations in L (1.2 GHz), C (5.3 GHz) and X (9.6 GHz) bands. However, the backscattering coefficients of the four canopies with different crown shapes and heights were simulated for canopies A and B at only VV and VH polarizations in the C (5.3 GHz) band with FEKO because of the long computational time and huge memory cost. The FEKO simulation establishes a good foundation to explore and develop applications of computational electromagnetic methods in microwave scattering domain of vegetation.
- The simulation results show that, for canopy A or B, different crown shapes possess significant differences in backscattering coefficients, of which the mean relative differences due to variations in crown shape are as high as 127%. Therefore, it can be demonstrated that the crown shape has a non-negligible influence on microwave backscattering coefficients of vegetation canopies. In turn, this also suggests that investigating the crown shape may have the potential to improve the simulation accuracy of microwave scattering models of vegetation, especially in canopies where volume scattering is the predominant mechanism.
- Regardless of whether canopy A’s or B’s parameters are set as model inputs, the backscattering coefficients of vegetation canopies with different crown shapes almost all gradually increase as the canopy height increases from 80 cm to 300 cm when simulated by either MTVM or FEKO. Taking MTVM for example, the exception is the cone canopy at VV, HV and VH polarizations in the L band (1.2 GHz) and at VV polarization in the X band (9.6 GHz), for which the MTVM simulation results when using canopy A’s parameters as inputs slightly decrease with the increase in crown height.
- For each specified model or method, the backscattering coefficients and their relative differences for canopy A are larger than those for canopy B for a given crown shape in the same band and polarization, which indicates that vegetation canopies with different components possess different backscattering characteristics. It also suggests that the crown shape effect acts synergistically with the effects of the vegetation component parameters, which mainly include the geometrical and physical parameters of the vegetation components.
- In preliminary experiments with MTVM and FEKO, a large discrepancy can be observed between the results of the three crown shapes studied and the reference cylinder. Overall, the ranking of the backscattering coefficients of the four vegetation canopies is $\sigma_{\mathrm{cone}}^{0}>\sigma_{\mathrm{cylinder}}^{0}>\sigma_{\mathrm{ellipsoid}}^{0}>\sigma_{\mathrm{inv}-\mathrm{cone}}^{0}$, which is approximately the same order of ranking as that of the volume fraction of the lower half of the vegetation canopy, for which the fractions are cone (75%) > cylinder (50%) = ellipsoid (50%) > inverted cone (25%). This correspondence can possibly be attributed to the attenuation effects of the upper canopy components; in other words, a lower volume fraction produces less attenuation, increasing the backscattering coefficients of the lower part of the canopy. However, a quantitative explanation of this result requires further analysis.
- Specifically, at VV and VH polarizations of the C band, the simulation results of FEKO are higher than those of MTVM. The reasons for the large difference may lie in different unreasonable assumptions and simplifications or the parameter settings of the two simulations. The actual reasons need to be further explored by analytical simulation and experimental measurement, which will be the focus of our future work.

## 5. Patents

Liu, L.; Li, S.J.; Xie, C.; Nie, J.; Shao, Y.; Yang, S.Q.; Xu, F.; He, H.X. A backscattering coefficients simulation method of GEO SAR for the vertical inhomogeneous vegetation canopy. China Patent No.106772362B, 3 May 2019.

## Figures and Tables

**Table 1:** Input parameters of canopy A.

Symbol	Value	Unit	Description
f	5.3	GHz	Radar Frequency
θ	43	Deg	Incidence Angle
H	80–300	cm	Canopy Height
$r_{\mathrm{leaf}}$	3	cm	Leaf Disc Radius
$d_{\mathrm{leaf}}$	0.02	cm	Leaf Disc Thickness
$w_{\mathrm{leaf}}$	0.85	100%	Leaf Volumetric Moisture
$r_{\mathrm{stalk}}$	1.25	cm	Stalk Cylinder Radius
$l_{\mathrm{stalk}}$	10	cm	Stalk Cylinder Length
$w_{\mathrm{stalk}}$	0.85	100%	Stalk Volumetric Moisture

**Table 2:** Input parameters of canopy B.

Symbol	Value	Unit	Description
f	5.3	GHz	Radar Frequency
θ	43	Deg	Incidence Angle
H	80–300	cm	Canopy Height
$r_{\mathrm{leaf}}$	0.3	cm	Needle Leaf Radius
$l_{\mathrm{leaf}}$	4	cm	Needle Leaf Thickness
$w_{\mathrm{leaf}}$	0.6	100%	Leaf Volumetric Moisture
$r_{\mathrm{branch}}$ ^1^	0.2	cm	Branch Cylinder Radius
$l_{\mathrm{branch}}$	30	cm	Branch Cylinder Length
$w_{\mathrm{branch}}$	0.6	100%	Branch Volumetric Moisture

^1^ For information about the grading of branches, refer to the Michigan Microwave Canopy Scattering Model (MIMICS) [3].

**Table 3:** The number of layers in the four canopies with different crown shapes and the number of vegetation components in each layer.

Item		Symbol	Canopy A	Canopy B
Layer Number		$N_{l}$	$\left\lfloor \frac{H}{l_{\mathrm{Stalk}}}+0.5\right\rfloor$	$\left\lfloor \frac{H}{l_{\mathrm{Branch}}}\right\rfloor$
Vegetation Component Number in *i*-th Layer	Cylinder	$N_{i}$	$\left\lceil \frac{\rho_{v}V_{h}}{N_{l}}\right\rceil$	$\left\lceil \frac{\rho_{v}V_{h}}{N_{l}}\right\rceil$
	Cone		$\left\lceil \rho_{v}V_{i}\right\rceil$	$\left\lceil \rho_{v}V_{i}\right\rceil$
	Inverted Cone			
	Ellipsoid			

**Table 4:** $\bar{\mu_{a}}$ and $\bar{\mu_{r}}$ of the simulation results between the three crown shapes studied and the reference cylinder crown shape using canopy A’s parameters as inputs.

Band	Polarization	Cone	Inverted Cone	Ellipsoid
L (1.2 GHz)	VV	1.32 ↓ ^1^	13.64	5.70
		12.13	126.68 ↑	53.05
	HH	3.16	13.76	4.89
		16.62	72.95	25.97
	VH	3.43	14.55 ↑	5.27
		11.68	50.09	18.20
	HV	3.36	14.45	5.23
		11.17 ↓	48.45	17.56
C (5.3 GHz)	VV	3.32	11.80	3.83
		17.14	61.25	19.93
	HH	3.59	11.78	3.60
		19.60	64.87 ↑	19.89
	VH	4.53	11.79	3.18
		16.01	42.02	11.39 ↓
	HV	4.80	11.82 ↑	3.17 ↓
		19.27	47.88	12.92
X (9.6 GHz)	VV	4.40	11.76 ↑	3.19
		15.65	42.75	11.61
	HH	4.52	11.70	3.15
		19.28	50.26 ↑	13.61
	VH	4.82	11.61	2.97 ↓
		16.02	38.93	9.99 ↓
	HV	4.81	11.66	2.97 ↓
		16.05	39.61	10.11

^1^ The notation ↑ denotes the maximum values of $\bar{\mu_{a}}$ and $\bar{\mu_{r}}$, and ↓ denotes the minimum values.

**Table 5:** $\bar{\mu_{a}}$ and $\bar{\mu_{r}}$ of the simulation results between the three crown shapes studied and the reference cylinder crown shape when using canopy B’s parameters as inputs.

Band	Polarization	Cone	Inverted Cone	Ellipsoid
L (1.2 GHz)	VV	3.76	11.60 ↑	3.45
		9.54	29.41 ↑	8.74
	HH	3.90	11.56	3.38
		7.85	23.29	6.80
	VH	5.65	11.10	2.38
		7.16	14.07	3.02
	HV	5.67	11.09	2.37 ↓
		6.90	13.50	2.88 ↓
C (5.3 GHz)	VV	2.63 ↓	13.74 ↑	5.01
		6.31 ↓	33.09	12.08
	HH	4.07	13.62	4.43
		10.35	34.61 ↑	11.27
	VH	5.28	13.48	3.76
		10.70	27.39	7.65
	HV	5.30	13.47	3.75
		9.83	25.02	6.97
X (9.6 GHz)	VV	2.59 ↓	11.70 ↑	3.98
		6.94	31.40	10.69
	HH	3.51	11.65	3.58
		9.89	32.90 ↑	10.11
	VH	4.89	11.51	2.86
		11.10	26.22	6.53
	HV	4.95	11.48	2.82
		10.36	24.11	5.94 ↓

**Table 6:** $\bar{\mu_{a}}$ and $\bar{\mu_{r}}$ of FEKO simulation results between the three crown shapes studied and the reference cylinder crown shape at VV and VH polarizations of C band (5.3 GHz).

Canopy	Polarization	Cone	Inverted Cone	Ellipsoid
A	VV	2.29	4.31	1.84 ↓
		16.17	31.65 ↑	13.50
	VH	2.34	5.76 ↑	2.52
		10.48 ↓	27.22	11.97
B	VV	2.70	4.17 ↑	2.37
		16.10	26.56 ↑	14.93
	VH	2.54	3.79	2.08 ↓
		12.18	19.71	10.77 ↓

**Table 7:** $\bar{\mu_{a}}$ and $\bar{\mu_{r}}$ of the MTVM simulation results for the same crown shapes between canopies A and B.

Polarization	Item	Cylinder	Cone	Inverted Cone	Ellipsoid
VV	$\bar{\mu_{a}}$	22.18 ↓	22.88	24.12 ↑	23.36
	$\bar{\mu_{rA}}$	115.14	143.28 ↑	77.53 ↓	101.01
	$\bar{\mu_{rB}}$	53.45	58.83 ↑	43.65 ↓	50.22
VH	$\bar{\mu_{a}}$	21.13	20.38 ↓	22.82 ↑	21.71
	$\bar{\mu_{rA}}$	75.06	86.12 ↑	57.03 ↓	69.18
	$\bar{\mu_{rB}}$	42.85	46.25 ↑	36.31 ↓	40.88

**Table 8:** $\bar{\mu_{a}}$ and $\bar{\mu_{r}}$ of the FEKO simulation results for the same crown shapes between canopies A and B.

Polarization	Item	Cylinder	Cone	Inverted Cone	Ellipsoid
VV	$\bar{\mu_{a}}$	2.71	2.55	2.47 ↓	3.09 ↑
	$\bar{\mu_{rA}}$	20.22	22.83 ↑	13.71 ↓	21.50
	$\bar{\mu_{rB}}$	15.90	17.48 ↑	11.72 ↓	16.55
VH	$\bar{\mu_{a}}$	2.38 ↓	3.54	4.59 ↑	3.25
	$\bar{\mu_{rA}}$	10.21 ↓	15.91	15.98 ↑	13.39
	$\bar{\mu_{rB}}$	12.59 ↓	20.15	20.66 ↑	16.21

## Data Availability

Not applicable.

## References

[1] Wang F., Tao J.J., Jiang L.M. (2011). Review of microwave remote sensing models of agricultural field. Remote Sens. Technol. Appl..

[2] Attema E.P.W., Ulaby F.T. (1978). Vegetation modeled as a water cloud. Radio Sci..

[3] Ulaby F.T., McDonald K., Sarabandi K., Dobson M.C. Michigan microwave canopy scattering models (MIMICS). Proceedings of the IEEE International Geoscience and Remote Sensing Symposium.

[4] Yueh S.H., Kong J.A., Jao J.K., Shin R.T., Le Toan T. (1992). Branching model for vegetation. IEEE Trans. Geosci. Remote Sens..

[5] Stiles J.M., Sarabandi K. (2000). Electromagnetic scattering from grassland—Part I: A fully phase-coherent scattering model. IEEE Trans. Geosci. Remote Sens..

[6] Bracaglia M., Ferrazzoli P., Guerriero L. (1995). A fully polarimetric multiple scattering model for crops. Remote Sens. Environ..

[7] Fu Y., Wang X.J., Sun X.J., Wang J. (2013). A study of tree crown information extraction method. World For. Res..

[8] Garestier F., Le Toan T. (2010). Forest modeling for height inversion using single-baseline InSAR/Pol-InSAR data. IEEE Trans. Geosci. Remote Sens..

[9] Jin Y.Q., Fei C. (2003). Scattering simulation for inhomogeneous layered canopy and random targets beneath canopies by using the Mueller matrix solution of the pulse radiative transfer. Radio Sci..

[10] Nelson R. (1997). Modeling forest canopy heights: The effects of canopy shape. Remote Sens. Environ..

[11] Calders K., Lewis P., Disney M., Verbesselt J., Herold M. (2013). Investigating assumptions of crown archetypes for modelling LiDAR returns. Remote Sens. Environ..

[12] Zhang N., Zhao Y.S. (2007). Effects of crown shape on reflectance of grassland. J. Remote Sens..

[13] Karam M.A., Fung A.K., Lang R.H., Chauhan N.S. (1992). A microwave scattering model for layered vegetation. IEEE Trans. Geosci. Remote Sens..

[14] Montomoli F., Brogioni M., Fontanelli G., Toccafonddi A., Lemmetyinen J., Pulliainen J., Hajnsek I., Macelloni G. Electromagnetic simulation and validation of backscattering from boreal forest in the C-Ku frequency range. Proceedings of the 2013 IEEE Geoscience and Remote Sensing Symposium.

[15] Monsivais-Huertero A., Sarabandi K., Chênerie I. (2010). Multipolarization microwave scattering model for Sahelian grassland. IEEE Trans. Geosci. Remote Sens..

[16] Thirion L., Chênerie I., Galy C. (2004). Application of a coherent model in simulating the backscattering coefficient of a mangrove forest. Waves Random Media.

[17] Ferrazzoli P., Guerriero L. (1996). Passive microwave remote sensing of forests: A model investigation. IEEE Trans. Geosci. Remote Sens..

[18] Ni W.J., Guo Z.F., Sun G.Q. (2010). Improvement of a 3D radar backscattering model using matrix-doubling method. Sci. Chin. Earth Sci..

[19] Liu L., Li S.J., Xie C., Nie J., Shao Y., Yang S.Q., Xu F., He H.X. (2019). A Backscattering Coefficients Simulation Method of GEO SAR for the Vertical Inhomogeneous Vegetation Canopy. China Patent.

[20] Altair F. (2020). Altair FEKO User Guide.

[21] Zhou Y.W., Sharma A., Kurum M., Lang R., O’Neill P., Cosh M. (2020). The backscattering contribution of soybean pods at L-band. Remote Sens. Environ..

[22] Burban L.L., Anderson J.W. (1994). Storms over the Urban Forest: Planning, Responding, and Regreening—A Community Guide to Natural Disaster Relief.

[23] Dente L., Ferrazzoli P., Su Z., Van Der Velde R., Guerriero L. (2014). Combined use of active and passive microwave satellite data to constrain a discrete scattering model. Remote Sens. Environ..

[24] Du J.Y., Shi J.C., Tjuatja S., Chen K.S. (2006). A combined method to model microwave scattering from a forest medium. IEEE Trans. Geosci. Remote Sens..

[25] Dong Y.F. (2005). Study on Rice Parameters Retrieval and Area Mapping with ENVISAT ASAR. Ph.D. Thesis.

[26] Jiang L.M. (2005). Passive Microwave Remote Sensing of Snow Water Equivalence Study. Ph.D. Thesis.

[27] Zhang G., Tsang L., Chen Z.X. (1996). Collective scattering effects of trees generated by stochastic lindenmayer systems. Microw. Opt. Technol. Lett..

[28] Liu L., Li K., Shao Y., Pinel N., Yang Z., Gong H.Z., Wang L.F. (2015). Extension of the monte carlo coherent microwave scattering model to full stage of rice. IEEE Geosci. Remote Sens. Lett..

[29] Shao Y., Li K., Brisco B., Liu L., Yang Z. The potential of polarimetric and compact SAR data in rice identification. Proceedings of the 35th International Symposium on Remote Sensing of Environment (ISRSE35).

[30] Xu M.S., Li K., Xie C., Zhu S., Luo H.Z., Zhang F.L., Wang X.J., Xia Z.S., Dang Y.F. (2014). Synergistically monitoring the snowstorm damaged forest with polarimetric SAR and optical remote sensing data. J. Nanjing For. Univ..

[31] Ulaby F.T., El-Rayes M.A. (1987). Microwave dielectric spectrum of vegetation—Part II: Dual-dispersion model. IEEE Trans. Geosci. Remote Sens..

[32] Levine D.M., Meneghini R. (1983). Scattering from arbitrarily oriented dielectric disks in the physical optics regime. J. Opt. Soc. Am..

[33] Eom H.J., Fung A.K. (1986). Scattering from a random layer embedded with dielectric needles. Remote Sens. Environ..

[34] Karam M.A., Fung A.K. (1988). Electromagnetic scattering from a layer of finite length, randomly oriented, dielectric, circular cylinders over a rough interface with application to vegetation. Int. J. Remote Sens..

[35] Brandewie A., Burkholder R.J. FEKO™ simulation of radar scattering from objects in low earth orbit for ISAR imaging. Proceedings of the 2020 International Applied Computational Electromagnetics Society Symposium (ACES, Virtual Conference).

[36] Herda D.L., Suryana J., Izzuddin A. Radar cross section of F35: Simulation and measurement. Proceedings of the 2020 6th International Conference on Wireless and Telematics (ICWT).

[37] Zhao J.J., Yin J.Y., Li C.F. (2013). RCS simulation of dihedral corner reflector based FEKO. Appl. Mech. Meter..

[38] Corbel C., Bourlier C., Pinel N., Chauveau J. (2013). Rough surface RCS measurements and simulations using the physical optics approximation. IEEE Trans. Antennas Propag..

[39] Wang X.F., Wang C., Liu Y. RCS computation and analysis of target using FEKO. Proceedings of the 2014 3rd Asia-Pacific Conference on Antennas and Propagation.

[40] Li T., Han H.B., Liu Y. (2015). Research on FEKO-based electromagnetic scattering characteristics of radar target. Mod. Electron. Tech..

[41] Liu L. (2015). A Coherent Microwave Scattering Model for Rice Field with Ear Based on Monte Carlo Simulation. Ph.D. Thesis.

